# Clinical Efficacy and Changes of Urothelial Dysfunction after Repeated Detrusor Botulinum Toxin A Injections in Chronic Spinal Cord-Injured Bladder

**DOI:** 10.3390/toxins8060164

**Published:** 2016-05-30

**Authors:** Sheng-Fu Chen, Chia-Hwei Chang, Hann-Chorng Kuo

**Affiliations:** Department of Urology, Buddhist Tzu Chi General Hospital and Tzu Chi University, 707, Section 3, Chung Yang Road, Hualian 970, Taiwan; madaux@yahoo.com.tw (S.-F.C.); cha615@tzuchi.com.tw (C.-H.C.)

**Keywords:** botulinum toxin A, urothelial dysfunction, spinal cord injury

## Abstract

Chornic spinal cord injury (SCI) will induce bladder urothelium dysfunction. This study investigated the therapeutic effects on urothelial dysfunction after repeated detrusor injections of onabotulinumtoxinA (BoNT-A) in SCI patients with neurogenic detrusor overactivity (NDO). Twenty chronic suprasacral SCI patients with NDO were enrolled. The patients received 300 U BoNT-A injection into the detrusor every six months. The urothelium was assessed by cystoscopic biopsy at baseline and six months after each BoNT-A treatment. Immunofluorescence staining for urothelial dysfunction, including E-cadherin, zonula occludens-1 (ZO-1), tryptase for mast cell activity, and urothelial apoptosis were investigated. The outcome of urothelial dysfunction parameters after BoNT-A injection were compared between baseline and six months after each treatment. Repeated 300 U BoNT-A injections showed a sustained decrease of detrusor pressure compared with baseline. After three repeated BoNT-A detrusor injections, significantly greater distributions of E-cadherin (*p* = 0.042) and ZO-1 (*p* = 0.003) expressions, but no significant changes, of urothelial apoptosis and mast cell activation were found after repeated BoNT-A therapy. Urothelial dysfunction, such as adhesive and junction protein concentrations in SCI patients’ bladders, recovered after three repeated cycles of BoNT-A treatment. The therapeutic effects sustained. However, urothelial inflammation and apoptosis after SCI were not significantly improved after three repeated BoNT-A injections.

## 1. Introduction

Spinal cord injury (SCI) is a significant cause of morbidity and mortality in developing countries, with the worldwide incidence of SCI reported in the literature ranges from 12.1 to 57.8 per million [1]. In chronic SCI patients, the main problems of neurogenic lower urinary tract dysfunction (NLUTD) are failure to store due to detrusor overactivity (DO), or urethral incompetence. Another problem is failure to empty due to detrusor areflexia, bladder neck dysfunction, or detrusor sphincter dyssynergia (DSD), and a usually-combined failure to store and empty, such as due to DSD or DO and impaired contractility [2]. If not well managed, high intravesical pressure may damage the upper urinary tract, causing renal scarring and chronic renal insufficiency, which greatly impairs the quality of life [3]. SCI also leads to rapid disruption of the uroepithelial barrier. This manifests in a loss of cell–cell interactions, decrease in transepithelial resistance, and increases in water and urea permeability [4]. Disruption of the urothelial barrier initiates a cascade of bladder dysfunction events, leading to suburothelial inflammation and vulnerability to chronic or recurrent cystitis or infection, or both. Suburothelial inflammation might also affect urothelial function, leading to a vicious cycle.

Our previous study had shown decreased expression of urothelial adhesive and junction proteins and increased suburothelial inflammation and apoptosis in patients with chronic SCI, regardless of injury level [5]. OnabotulinumtoxinA (BoNT-A) has been proved useful in the treatment of NDO in SCI patients in a large phase-three clinical trial [6].

BoNT-A is one of the most powerful neurotoxins; it inhibits the release of neurotransmitters from the nerve fibers and urothelium [7]. It is possible to stem the disruption of the uroepithelium by modulating the release of neurotransmitters and inflammatory mediators. BoNT-A injection decreases bladder nerve growth factor (NGF) production and urinary NGF levels, suggesting an anti-inflammatory response can be achieved after intravesical BoNT-A treatment [8]. In our recent study, the urothelial dysfunction and adhesive and junction protein concentrations in SCI patients’ bladders recovered after BoNT-A treatment after a single injection [9].

Repeated BoNT-A detrusor injection has been shown to have good results and safety for control of NDO in chronic SCI bladders [10,11]. However, there is insufficient data to definitively determine whether repeated BoNT-A injection into the detrusor has a sustained improvement in the urothelial dysfunction. This study investigated the therapeutic effects of repeated detrusor injection of BoNT-A on urothelial dysfunction in SCI bladders.

## 2. Results

A total of 20 patients with chronic SCI and 10 controls were enrolled in this study. The SCI patients included 11 men and nine women with a mean age of 48.2 ± 18.41 years. Among them, 15 had complete injuries while five had incomplete injuries. The injury level was at cervical spinal cord in seven patients, thoracic cord in 11, and lumbar cord in two. Among the SCI patients, 16 patients had DSD. No patient having an indwelling catheter or cystostomy was enrolled in this study. The control group included 10 women with stress urinary incontinence with a mean age of 52.40 ± 10.51 years.

The changes in urodynamic parameters after repeated BoNT-A injections are shown in Table 1. Significant increases in cystometric bladder capacity (CBC) and post-void residual (PVR) volume, and a significant decrease in detrusor pressure at Qmax (Pdet.Qmax) were noted six months after each BoNT-A injection. The therapeutic effects of repeated injections on the objective measures of Video-urodynamic study (VUDS) were consistent throughout the treatment course until the end of the study. The Pdet.Qmax decreased to less than 25 cmH_2_O, which was considered a level safe enough to prevent upper urinary tract damage. Not surprisingly, the PVR volume also increased along with the incremental CBC increase.

**Figure 1:**
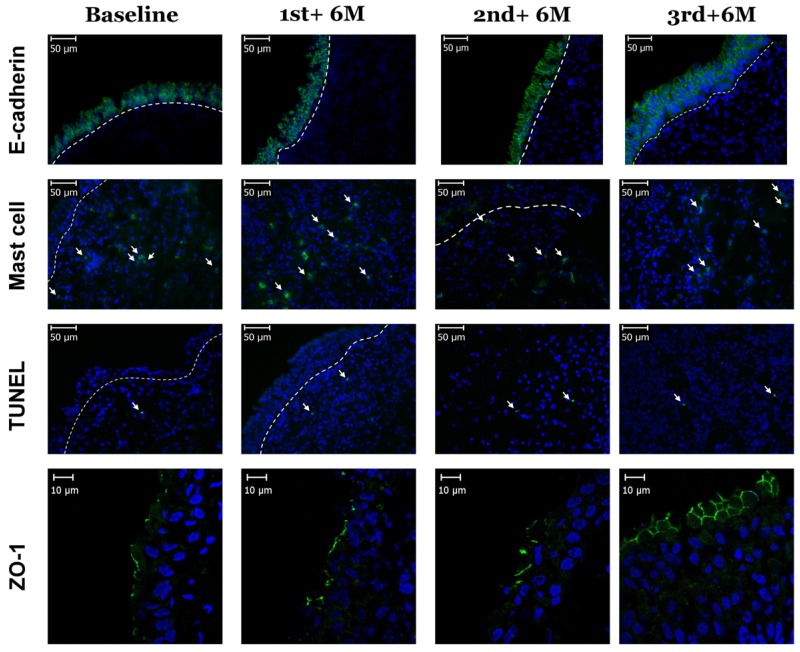
Shows the significantly lower expressions of E-cadherin and zonula occludens-1 (ZO-1) in the spinal cord injury (SCI) bladders compared with the corresponding expressions in the control bladders. The target cells are labelled in green (as the arrows indicate) and the white dotted lines indicate the boundary between the urothelium and suburothelium.

Table 2 showed the changes of the urothelial dysfunction at baseline and six months after each set of 300 U BoNT-A detrusor injections and the control. At baseline, the expression of E-cadherin, ZO-1 in the SCI patients decreased significantly, compared with the controls (*p* < 0.01). A significant increase in urothelial cell apoptosis was also observed in the bladder specimens from SCI patients (*p* = 0.047). Figure 1 shows the significantly lower expressions of E-cadherin and ZO-1 in the SCI bladders compared with the corresponding expressions in the control bladders (*p* < 0.001).

At six months after each BoNT-A injection, persistent improvements of E-cadherin and ZO-1 expressions were noted compared with the baseline. After the third BoNT-A injection, the distribution of E-cadherin expression was significantly higher compared with baseline (42.4 ± 27.0 *vs.* 25.6 ± 21.4 fluorescence intensity units per 4 μm^2^, *p* = 0.004). The same trend was also noted in ZO-1 expression. A significantly higher distribution of ZO-1 after repeated second and third BoNT-A injection compared with baseline was noted (6.86 ± 7.25 *vs.* 2.77 ± 3.24, *p* = 0.008, and 8.32 ± 6.78 *vs.* 2.77 ± 3.24, *p* = 0.003, respectively). However, the mast cell activity showed no significant difference between baseline and six months after each injections, nor did urothelial cell apoptosis differ significantly after repeated BoNT-A treatment compared with baseline (Table 2).

When we compared the urothelial dysfunction parameters after repeated BoNT-A injection with the controls the distribution of E-cadherin expression improved to non-significant difference from the control after the first and second BoNT-A injections, and sustained at six months after the third BoNT-A injection. (*p* = 0.187, *p* = 0.403, *p* = 0.812, respectively). The same finding was also noted in ZO-1 expression (*p* = 0.11, *p* = 0.965, *p* = 0.373, respectively). On the other hand, the urothelial cell apoptosis did not decrease after each BoNT-A injection and showed persistently greater than the control (*p* = 0.03, *p* = 0.03, *p* = 0.04, respectively).

No severe adverse events such as general weakness or severe autonomic dysreflexia occurred after BoNT-A injection. Five patients had slight hematuria which was spontaneously resolved within three days without intervention. During the repeated BoNT-A treatment course, one-third patients suffered from symptomatic urinary tract infection (UTI) (febrile or micturition pain with pyuria and positive urine culture), which were carefully controlled by antibiotic treatment. The rate of satisfactory dryness reported by patients was 60%.

## 3. Discussion

This study shows that impaired expression of the adhesive protein E-cadherin and tight junction protein ZO-1 and significantly higher apoptosis in the urothelium of patients with chronic SCI and NDO. After repeated detrusor BoNT-A injections, E-cadherin, and ZO-1 expressions were recovered, and the effects sustained up to 6 months after the third treatment. However, the chronic inflammation and urothelial apoptosis did not improve after repeated BoNT-A injections.

To our knowledge, there is limited data focus on urothelial dysfunction after repeated detrusor BoNT-A injections for SCI patients with NDO. This study provides evidence that repeated detrusor BoNT-A injections improve the impaired adhesive and junction proteins of urothelium in SCI patients. Lower urinary tract dysfunction and complications are major concerns in the management of SCI patients. Apodaca *et al.* found that SCI also led to rapid disruption of the uroepithelial barrier [4]. The impaired urothelium in SCI bladders may cause chronic infammation and easy to induce UTI. SCI is associated with a number of changes in the urothelium, including both structural changes and defects in urothelial signaling changes [12]. Our previous study showed increased urothelial cell apoptosis and chronic inflammation are associated with recurrent UTI [13]. The asymptomatic bacteriuria might result in increased excitability of suburothelial sensory fibers and induce DO at a small bladder volume. Cruz *et al.* recently proposed that the changes in the urothelium after SCI might play a role in the pathophysiology of NDO [14].

Since 2000, minimally invasive BoNT-A injections into the detrusor have been reported to improve clinical and urodynamic parameters and quality of life in patients with refractory NDO in several open-label studies [15,16,17,18,19]. In our previous study, the success rate was 73.3% in SCI patients with NDO who received a single detrusor injection of 200 U BoNT-A, and the therapeutic effect lasted for 3 to 9 months (mean, 5.3 months) [20]. Based on that study, the injection protocol in this study was designed as repeated injections every six months in order to observe the consistency of therapeutic effects of the repeated BoNT-A injections.

BoNT-A can inhibit adenosin triphosphate release from the urothelium [7]. BoNT-A treatment not only inhibits transmitter release from efferent nerve endings but also sensory nerve terminals and urothelium [7]. In humans with NDO the expressions of bladder muscarinic receptors M2 and M3 and purinergic receptors P2X_2_ and P2X_3_ are reduced after detrusor BoNT-A injections, suggesting that BoNT-A inhibits DO by acting through inhibition of both the sensory and motor arms of the micturition reflex [21,22,23,24].

E-cadherin is a calcium-dependent glycoprotein, which plays a critical role in cell-to-cell adhesion. Loss of E-cadherin function can trigger cancer progression and metastasis [21]. ZO-1 is a tight junction protein that maintains the highly resistant urothelial barrier [25]. Previous study has also established a molecular connection between E-cadherin and transient receptor potential vanilloid 4 channel (TRPV4), suggesting that E-cadherin is also associated with bladder sensation and barrier function [26]. Our previous study showed decreased expression of E-cadherin and junction protein ZO-1 in patients with chronic SCI, regardless of injury level [5]. In the current study, the urothelial barrier function recovered after BoNT-A injections by improving the adhesive and tight junction protein levels. This result might, in part, explain the mechanism of decrease of the hyperexcitability of C-fiber bladder afferents, thereby reducing NDO. Our recent study reported the urothelial dysfunction improved at short-term but not persistent in a single BoNT-A injection [9]. This study further provides evidence that repeated BoNT-A injections can have a sustained therapeutic effect on NDO in SCI patients.

The mast cells degranulate and release pre-formed mediators of inflammation including histamine, heparin, and tryptase [27]. Apoptosis is a stepwise process characterized by a series of stereotypical morphological changes that eventually lead to cell death. Urothelial cell apoptosis in patients with interstitial cystitis/bladder pain syndrome resulted from upregulation of the inflammatory signals, including p38 mitogen-activated protein kinase and tumor necrosis factor-α [28]. In the present study, we showed that the apoptotic process was highly activated in the bladders of patients with SCI compared to the control group. After repeated BoNT-A injections, the urothelial apoptosis and mast cell activity were persistently higher than controls, which suggested that the neurogenic inflammation of SCI bladders cannot be inhibited by BoNT-A treatments. One possible cause for this result is the presence of asymptomatic UTI in one third of patients during the BoNT-A treatment course. Despite of this, the barrier function improvement could prevent the vicious cycle which might lead to end-stage bladder dysfunction.

In addition to the benefit of decreased detrusor pressure to prevent renal damage, BoNT-A injection for SCI with NDO significantly improves quality of life [29]. In the past 10 years, most study used 300 U of BoNT-A for treatment of NDO, usually as 30 injections of 10 U/mL in the bladder (excluding the trigone) under cystoscopic guidance and with different types of anesthesia [16]. Several phase-three multicenter trials were conducted to investigate the efficacy and safety of detrusor BoNT-A injections in patients with NDO due to SCI or multiple sclerosis. BoNT-A 200 U or 300 U injection significantly reduced urinary incontinence episodes compared with placebo. The effects were equally observed in both SCI and multiple sclerosis patients, and less side effects occurred in 200 U treatment group [6]. This study shows that BoNT-A injection improves NDO in SCI bladders not only by anticholinergic effect, but also by the improvement of the urothelium function after treatment.

Our results showed the barrier function was improved after BoNT-A treatment, but the urothelial apoptosis and mast cell activation expression did not improve after repeated BoNT-A injections. The cause why the discrepancy occurs after BoTN-A injections remained further study. We speculated that the bladder biopsy was taken at six months after the latest BoNT-A injection, which might lower the therapeutic efficacy of BoNT-A on neurogenic inflammation due to SCI. In addition, the presence of asymptomatic UTI in one third of patients during the BoNT-A treatment course might also have affect on this result. Put together, although BoNT-A injection can improve barrier function in SCI bladders, the increased suburothelial inflammation and urothelial apoptosis did not show significant improvement after three BoNT-A injections. Nevertheless, the persistent effects of BoNT-A on increase of bladder capacity and decrease of detrusor pressure are beneficial to the SCI patients with refractory urinary incontinence.

A limitation of the present study is the small case number and short term follow-up. Secondly, around one-third of patients had asymptomatic UTI during follow-up might affect the results of anti-inflammatory effects of BoNT-A. Another limitation is that we did not analyze the patients smoking history, which might affect the urothelial function. Finally, there is no histologic assessment, which may provide compatible information to support our results. In addition, the therapeutic effects of sensory protein improvements of TRPV1, P2X receptors, connexin, and endothelial nitric oxide synthase have not yet been thoroughly investigated and require further study. Despite of these limitations, this preliminary study has demonstrated the therapeutic effect of BoNT-A treatment on improvement of urothelial dysfunction in the chronic SCI bladders.

## 4. Conclusions

Urothelial dysfunction and adhesive and junction protein concentrations in SCI bladders recovered after repeated 300 U BoNT-A injections. The therapeutic effects sustained up to six months after the third BoNT-A injection and reach the normal level. However, neurogenic inflammation and urothelial apoptosis after SCI was not significantly improved after repeated BoNT-A injections.

## 5. Materials and Methods

In this prospective study, 20 patients with chronic SCI and NDO refractory to anti-muscarinic treatment were consecutively enrolled. All patients received four sets of 300 U of BoNT-A (Allergan, Irvine, CA, USA) detrusor injection every six months. VUDS was routinely performed prior to enrolment to confirm the presence of DO with or without DSD. Patients were excluded if they had an active UTI or urinary tract cancer at enrolment, history of lower urinary tract surgery, or chronic systemic diseases such as congestive heart failure and chronic renal failure. Additionally, repeated BoNT-A injection would be hold when patients were found to have active UTI before treatment. All patients had been educated to perform intermittent catheterization (IC) due to possible urinary retention after BoNT-A injection. IC is the standard management in European Association of Urology (EAU) guideline for NLUTD patients who are unable to empty their bladder adequately [30].

All subjects gave their informed consent for inclusion before they participated in the study. The study was conducted in accordance with the Institutional Review Board, and the protocol was approved by the Ethics Committee of Buddhist Tzu Chi General Hospital (IRB: TCGH 101-61, 30 October 2016). Each patient was informed about the study rationale and procedures, and written informed consent to participate in the study was obtained before any bladder procedure. The patients received BoNT-A injection under intravenous general anesthesia in the operating room. Each vial of BoNT-A (100 U) was diluted with 10 mL of normal saline and 30 injections evenly distributed into the detrusor were given using a 23-gauge needle (Richard Wolf, Knittlingen, Germany) and a rigid cystoscopic injection instrument (22 French Richard Wolf, Knittlingen, Germany). Four bladder cold cup biopsies were taken randomly at the posterior wall above the interureteric ridge, and only the bladder mucosa and submucosa were taken to prevent bladder perforation. One bladder biopsy specimen was sent to the pathology department for hematoxylin and eosin staining to exclude the possibility of carcinoma *in situ*. The remaining three specimens were stored in optimum cutting temperature (OCT) and liquid nitrogen for further immunohistochemistry studies.

At six months after the previous BoNT-A treatment, patients underwent repeat VUDS and bladder biopsy and then received the next BoNT-A injection at that time. We also invited 10 female patients with stress urinary incontinence but without urgency frequency symptoms to serve as controls for comparison. All control patients had been proven free of bladder outlet obstruction by VUDS. The bladder tissue was obtained during anti-incontinence surgery and processed as the SCI patients. Male patient was not selected to serve as the controls, because the prostate might cause bladder outlet obstruction and affect the urothelial function [31].

Immunofluorescence staining and quantification of protein expression in the bladder tissues of SCI patients and controls were investigated for urothelial adhesive and junction proteins by measuring E-cadherin expression and ZO-1 expression, respectively. Mast cell activation was assessed by measuring tryptase levels. Urothelial cell apoptosis was evaluated using the terminal deoxynucleotidyl transferase dUTP nick end labelling (TUNEL) assay as described previously [21,32].

The urinary bladder specimens were immersed and fixed for 1 h in an ice-cold solution of 4% formaldehyde in phosphate buffered saline (PBS) (pH 7.4) (SIGMA, Saint Louis, MO, USA). They were then rinsed with ice-cold PBS containing 15% sucrose (SIGMA, Saint Louis, USA) for 12 h. Biopsy specimens were embedded in OCT medium and stored at −80 °C. Four sections per specimen were cut using a cryostat HM550 OP (Microm International GmbH, Walldorf, Germany) at a thickness of 8 μm and collected on new silane III-coated glass slides (Muto Pure Chemicals Co. Ltd., Tokyo, Japan). Sections were post-fixed in acetone (SIGMA, Saint Louis, MO, USA) at −20 °C and blocked with Power Blocking reagent 10× (ten multiplication) (BioGenex, Fremont, CA, USA). The sections were incubated overnight at 4 °C with primary antibodies to anti-human E-cadherin (BD Biosciences, San Jose, CA, USA) or anti-human tryptase (Chemicon, Temecula, CA, USA). After rinsing the sections with 0.1% Tween-20 in BPS, rabbit anti-mouse conjugated fluorescein isothiocyanate secondary antibodies (DakoCytomation Denmark A/S, Glostrup, Denmark) were applied to the sections and incubated for 1 h. Finally, the sections were counterstained with 4',6-diamidino-2-phenylindole (DAPI; Sigma Chemical Co., Saint Louis, MO, USA). Negative controls included the isotype of the primary antibody. We obtained the mean, maximum, range and standard deviation (SD) values of the staining intensity and the percent positive area measurements using three random hot spots within each specimen. The expression of E-cadherin and ZO-1 in the urothelium was quantified using ImageJ software, developed by the National Institutes of Health (Bethesda, MD, USA) [33]. Immunofluorescence (tryptase and TUNEL assays) was quantified by counting the number of positively stained cells/total cells per unit area (4 μm^2^). The results were shown as the percentage of positive cells per 100 total cells. Differences in the expressions of proteins in the urothelium at baseline and 6 months after each BoNT-A injection were analyzed using the paired *t*-test. All calculations were performed using SPSS for Windows, version 16.0 (SPSS, Chicago, IL, USA). *p* < 0.05 was considered to indicate statistical significance.

## Figures and Tables

**Table 1:** Changes of the urodynamic parameters at 6 months after each set of 300 U BoNT-A detrusor injection compared with baseline and the control.

Videourodynamic Parameter	Control	Baseline 1st BoNt-A	1st BoNT-A + 6 M	2nd BoNt-A + 6 M	3rd BoNT-A + 6 M
CBC (mL)	364 ± 196	163 ± 97.7 *	223 ± 150 **	245 ± 137 **	239 ± 118 **
Pdet(cmH_2_O)	25.2 ± 14.5	42.7 ± 12.5 *	33.2 ± 28.6 **	22.8 ± 16.9 **	22.2 ± 19.1 **
Qmax (mL/s)	11.9 ± 6.25	6.9 ± 6.27 *	2.61 ± 3.50 **	2.75 ± 4.89 **	3.15 ± 4.97 **
Volume (mL)	302 ± 154	91.4 ± 99.2 *	34.9 ± 51.3 **	38.6 ± 71.5 **	57.7 ± 82.6 **
PVR (mL)	93.3 ± 107	118 ± 90.7 *	245 ± 179 **	343 ± 190 **	329 ± 170 **
Compliance (mL/cmH_2_O)	86.3 ± 38.0	25.6 ± 28.3 *	30.0 ± 35.1	36.7 ± 32.5	44.5 ± 56.2

CBC: cystometric bladder capacity; Pdet: detrusor pressure at Qmax; Qmax: maximum flow rate; PVR: post-void residual volume; BoNT-A: OnabotulinumtoxinA. Data are expressed as mean ± standard deviation. * Indicates significantly different between control and the baseline before BoNT-A treatment of SCI bladders; ** Indicates significant difference between six months after each BoNT-A treatment compared with baseline.

**Table 2:** Changes of the urothelial dysfunction parameters at baseline and 6 months after each set of 300 U BoNT-A detrusor injections and compared with control.

Urothelial Dysfunction Parameter	Control	Baseline 1st BoNT-A	1st BoNT-A + 6 M	2nd BoNT-A + 6 M	3rd BoNT-A + 6 M
E-cadherin	41.3 ± 8.40	25.6 ± 21.4 *	29.5 ± 28.4	30.9 ± 32.8	42.4 ± 27.0 ^#^
Mast cell	5.89 ± 4.92	7.51 ± 6.60	9.61 ± 6.92	7.47 ± 6.22	9.87 ± 7.29
TUNEL	1.00 ± 1.35	2.40 ± 4.67 *	2.59 ± 2.11 *	2.23 ± 1.31 *	2.42 ± 2.45 *
ZO-1	6.37 ± 1.72	2.77 ± 3.24 *	5.08 ± 6.09	6.86 ± 7.25 ^#^	8.32 ± 6.78 ^#^

TUNEL: the terminal deoxynucleotidyl transferase dUTP nick end labelling; ZO-1: zonula occludens-1. Data are expressed as mean ± standard deviation. * Indicates significantly different (*p* < 0.05) between each BoNT-A treatment compared with control group; ^#^ Indicates significantly different (*p* < 0.05) 6 months after each BoNT-A treatment compared with baseline.
